# Polymer-Based Microencapsulation of *Hedychium coronarium* Rhizome Essential Oil for Enhanced Bioactivity Stability and Reduced Irritation

**DOI:** 10.3390/pharmaceutics18040443

**Published:** 2026-04-03

**Authors:** Pattiya Tammasorn, Wannaree Charoensup, Watchara Kanjanakawinkul, Wei-Chao Lin, Thomas Rades, Wantida Chaiyana

**Affiliations:** 1 Department of Pharmaceutical Sciences, Faculty of Pharmacy, Chiang Mai University, Chiang Mai 50200, Thailand; pattiya_tammasorn@cmu.ac.th (P.T.); wannaree.charoensup@cmu.ac.th (W.C.); 2 Chulabhorn Royal Pharmaceutical Manufacturing Facilities by Chulabhorn Royal Academy, Chon Buri 20180, Thailand; watchara.kan@cra.ac.th; 3 Department of Cosmetic Science and Institute of Cosmetic Science, Chia Nan University of Pharmacy and Science, Tainan 71710, Taiwan; weilin@mail.cnu.edu.tw; 4 Department of Pharmacy, University of Copenhagen, Universitetsparken 2, 2100 Copenhagen, Denmark; thomas.rades@sund.ku.dk; 5 Research Center of Pharmaceutical Nanotechnology, Chiang Mai University, Chiang Mai 50200, Thailand; 6 Research Center of Deep Technology in Beekeeping and Bee Products for Sustainable Development Goals (SMART BEE SDGs), Chiang Mai University, Chiang Mai 50200, Thailand; 7 Multidisciplinary and Interdisciplinary School, Chiang Mai University, Chiang Mai 50200, Thailand

**Keywords:** butterfly ginger, essential oil, eucalyptol, natural polymers, encapsulation, irritancy reduction, bio-based materials, sustainable formulation, plant-derived bioactive

## Abstract

**Background**: Plant-derived essential oils possess valuable bioactivities, but their application is limited by volatility and irritation, which may be addressed through natural polymer encapsulation. This study aimed to investigate the bioactivity of *Hedychium coronarium* rhizome essential oil and evaluate the effect of microencapsulation on its physicochemical characteristics, biological stability, and irritation profile. **Methods**: Essential oil was extracted from *H. coronarium* rhizomes by hydrodistillation and chemically characterized. Enzyme inhibitory activities against elastase, hyaluronidase, and tyrosinase were assessed. Microencapsulation was performed using gum Arabic or maltodextrin at 1–5% *w*/*w* oil loadings. The resulting powders were evaluated for morphology, entrapment efficiency, hygroscopicity, water activity, biological stability, and irritation potential using the hen’s egg test on the chorioallantoic membrane. **Results**: The essential oil demonstrated strong enzyme inhibition, particularly against hyaluronidase (IC_50_ = 0.1 ± 0.0 µg/mL), along with notable elastase and tyrosinase inhibition. Encapsulation significantly reduced irritation scores from 13.3 ± 1.4 for the free oil to 3.6–4.2 for encapsulated systems (*p* < 0.05). Gum Arabic produced rough, porous particles with lower hygroscopicity, while maltodextrin yielded smoother particles with lower water activity. Both encapsulated powders significantly enhanced biological stability compared with the ethanolic solution. **Conclusions**: Natural polymer-based microencapsulation effectively reduced the irritation potential and improved the handling properties of *H. coronarium* essential oil, supporting its potential application in topical bioactive delivery systems.

## 1. Introduction

Plants have long provided a rich source of bioactive compounds for various medicinal and cosmetic uses [1,2,3], with essential oils having gained particular attention, due to their reported antimicrobial, antioxidant, and enzyme inhibitory activities [4,5]. These features have placed essential oils as promising active ingredients in cosmeceutical formulations for skin health maintenance and the retardation of skin aging [5].

*Hedychium coronarium* J. Koenig, commonly called white ginger lily, is a perennial rhizomatous herbaceous plant belonging to the family Zingiberaceae, native to Southeast Asia and widely cultivated in tropical and subtropical areas. *H. coronarium* has been traditionally valued for its aromatic rhizomes and flowers for use in folk medicine because of its anti-inflammatory, analgesic, and antimicrobial properties [6,7]. The rhizomes of *H. coronarium* are particularly rich in essential oils composed primarily of monoterpenoids and sesquiterpenoids [8]. These terpenoid compounds have been associated with a range of biological activities that are relevant to skin health, particularly in relation to skin aging, pigmentation, and oxidative protection [8,9]. Such effects are frequently investigated through the inhibition of key skin-related enzymes, a strategy that has been widely applied in studies of plant-derived extracts and essential oils [10,11,12]. Enzymes such as elastase, hyaluronidase, and tyrosinase play critical roles in skin aging and pigmentation [10,11,12]. Elastase degrades elastin fibers, leading to loss of skin elasticity and the formation of wrinkles [13,14], whereas hyaluronidase breaks down hyaluronic acid, a key molecule in skin hydration and structural integrity [15,16]. Tyrosinase is the rate-limiting enzyme in melanin biosynthesis, and its overactivity can result in hyperpigmentation [8,12]. The inhibition of these enzymes has therefore emerged as an effective strategy for anti-aging and skin-whitening formulations. Previous studies have shown that essential oils can exert significant anti-elastase, anti-hyaluronidase, and anti-tyrosinase activities [12,17].

Despite their promising biological activities, the direct use of essential oils in topical applications is often limited by several factors, including volatility, chemical instability, and potential skin irritation [18,19]. Many essential oils can cause redness, burning, or allergic reactions upon direct application, which restricts their utility in cosmetic formulations [20,21,22]. Ensuring dermal safety while retaining bioactivity is therefore a major challenge in the development of essential oil-based cosmeceuticals. Recent advances in formulation technologies have highlighted encapsulation as a promising approach to address these limitations [23].

Encapsulation involves the entrapment of active compounds within a carrier matrix, such as natural polymers, which can protect sensitive bioactive compounds from degradation, enhance water solubility, and control release [24]. In addition, encapsulation can markedly reduce the irritancy potential of essential oils, making them safer for dermal application while maintaining or even enhancing their bioactivity [18,19]. Natural polymers such as gum Arabic and maltodextrin have been widely employed for encapsulation due to their biocompatibility, low toxicity, and ability to form stable matrices for essential oils [25]. Encapsulation of essential oils using these polymers not only facilitates handling and incorporation into cosmetic formulations but also offers a means to reduce irritation, which is critical for sensitive skin applications [26].

Therefore, this study aimed to investigate the chemical composition and cosmeceutical bioactivity of *H. coronarium* rhizome essential oil, with a particular focus on anti-elastase, anti-hyaluronidase, and anti-tyrosinase activities. Additionally, the study evaluated the effectiveness of encapsulation using natural polymers, including gum Arabic and maltodextrin, in reducing the irritancy of the essential oil for cosmetic applications. The findings are expected to contribute to the development of novel, plant-based formulations that harness the effects of essential oil components while minimizing adverse effects, ultimately supporting the growing demand for natural and safe cosmetic products.

## 2. Materials and Methods

### 2.1. Plant Materials

Fresh rhizomes of *H. coronarium* were collected from the agricultural field of the Chulabhorn Royal Pharmaceutical Manufacturing Facility, Chonburi, Thailand, in November 2022. Species identification was confirmed by Miss Wannaree Charoensup, a botanist at the Department of Pharmaceutical Sciences, Faculty of Pharmacy, Chiang Mai University, Thailand, based on morphological examination of the whole fresh plant, including its flowers, leaves, and rhizomes. A voucher specimen No. 0023314 was deposited in the herbarium of the Faculty of Pharmacy, Chiang Mai University, Thailand. The rhizomes were thoroughly cleaned to remove dirt and soil, manually sliced into small pieces, and dried in a hot air oven (Memmert, Schwabach, Germany) set at 45 °C for 72 h. The dried material was then ground into a fine powder using an electric blender (Sharp Thai Co., Ltd., Bangkok, Thailand) and stored in a tightly sealed container for subsequent experiments. A small quantity of the dried rhizome powder was mounted on a glass slide using diluted glycerol and examined under a Nikon ECLIPSE E200 microscope (Nikon Solutions Co., Ltd., Konan, Japan) equipped with a Canon EOS 750D camera (Canon Inc., Tochigi, Japan). Images were captured at 40× magnification and identified by Miss Wannaree Charoensup, a botanist at the Department of Pharmaceutical Sciences, Faculty of Pharmacy, Chiang Mai University, Thailand.

### 2.2. Chemical Materials

Eucalyptol (≥98.0%) was standard grade purchased from Tokyo Chemical Industry Co., Ltd. (Tokyo, Japan). Elastase from porcine pancreas (EC 3.4.21.36, ≥4.0 units/mg protein), hyaluronidase from bovine testes (EC 3.2.1.35, lyophilized powder, 400–1000 units/mg solid), lyophilized tyrosinase from mushroom (EC 1.14.18.1, lyophilized powder, ≥1000 unit/mg solid), n-succinyl-Ala-Ala-Ala-p-nitroaniline (AAAPVN, ≥98.0%), hyaluronic acid, L-tyrosine (≥98.0%), L-3,4-dihydroxyphenylalanine (L-DOPA, ≥98.0%), kojic acid (≥98.5%), epigallocatechin gallate (EGCG, ≥95.0%), anhydrous sodium sulfate, and oleanolic acid (≥97.0%) were purchased from Sigma-Aldrich (Schnelldorf, Germany). Bovine serum albumin (BSA) was purchased from Merck (Darmstadt, Germany). Tris (hydroxymethyl) aminomethane (Tris base), hydrochloric acid (HCl, 37%), acetic acid (CH_3_COOH, ≥99.7%), sodium chloride (NaCl), monosodium phosphate (NaH_2_PO_4_), disodium phosphate (Na_2_HPO_4_), and deionized (DI) water were purchased from RCI Labscan (Bangkok, Thailand). Dimethyl sulphoxide (DMSO, ≥99.7%), n-hexane (≥99%), and ethanol (≥99.5%) were analytical grade purchased from RCI Labscan (Bangkok, Thailand). Gum Arabic and maltodextrin were cosmetic-grade purchased from Krungthepchemi Co., Ltd. (Bangkok, Thailand). Acetonitrile (≥99.9%) was HPLC grade purchased from Fisher Scientific (Cleveland, OH, USA).

### 2.3. Extraction of Essential Oils from H. coronarium Rhizomes

The essential oil from *H. coronarium* rhizomes was extracted using a hydrodistillation method [27]. The dried powder of *H. coronarium* rhizome was soaked in DI water at a weight-to-volume ratio of 1:20 and placed into a distillation flask positioned in a heating mantle (MS-E107, MTOPS Co., Ltd., Suwon, Republic of Korea). Hydrodistillation was performed using a Clevenger apparatus (Schott Duran, Schott AG, Mainz, Germany). Once the vaporization commenced, the distillation was continued for 2 h. After completion, heating was discontinued and the essential oil was allowed to cool to room temperature. Residual moisture was removed by treating the oil with anhydrous sodium sulfate. The essential oil was then collected and stored in a light-protected container at 4 °C.

### 2.4. High-Performance Liquid Chromatography (HPLC) Analysis

HPLC analysis was conducted using a D-7420 chromatography system (Hitachi High-Technologies Corporation, Tokyo, Japan) equipped with a UV detector set at a wavelength of 200 nm. Separation was achieved on a reversed-phase HC-C18 column (4.6 × 250 mm internal diameter, 5 μm particle size; Agilent Technologies, Santa Clara, CA, USA). The chromatographic conditions employed an isocratic mobile phase composed of acetonitrile and DI water in a 7:3 ratio, delivered at a constant flow rate of 1.0 mL/min. An injection volume of 20 μL was used for the *H. coronarium* essential oil samples and their reference compound (eucalyptol). Quantitative determination of *H. coronarium* rhizome essential oils was performed using eucalyptol as the reference compound due to its prominence and relevance as a major constituent of *H. coronarium* essential oil [27,28,29]. The HPLC method was partially validated in terms of linearity, sensitivity, and repeatability to ensure its suitability for the quantification of eucalyptol. A calibration curve for eucalyptol was constructed at five concentration levels ranging from 5 to 50 μg/mL to generate a reliable calibration curve with good linearity (R^2^ = 0.9999). The limit of detection (LOD) and limit of quantification (LOQ) were calculated based on signal-to-noise ratios of 3 and 10, respectively, in accordance with the ICH guidelines [30], and were determined to be 1.03 and 3.12 μg/mL, respectively. Essential oil samples were diluted to a concentration of 100 μg/mL prior to injection to fall within the calibration range. All assessments were performed in triplicate.

### 2.5. Biological Activity Determination

#### 2.5.1. Elastase Inhibitory Activities

The elastase inhibitory activity of *H. coronarium* rhizome oils was evaluated following the method of Thring et al. (2009) [31]. Initially, elastase enzyme activity was determined and only the enzyme preparations exhibiting greater than 90% activity were utilized in the assay. The sample was mixed with porcine pancreatic elastase solution at a ratio of 1:4 and incubated for 15 min at room temperature. The enzymatic reaction was initiated by adding 100 µL of N-succinyl-Ala-Ala-Ala-p-nitroanilide (AAAPVN) substrate dissolved in Tris-HCl buffer pH 8.0. The reaction progress was continuously monitored for 20 min at 410 nm using a multimode microplate reader (BMG Labtech GmbH, Ortenberg, Germany). To minimize potential assay interference caused by essential oil components (e.g., turbidity or intrinsic absorbance), both blank of sample (sample in buffer without enzyme and substrate) and blank of control (sample solvent in buffer without enzyme and substrate) were included and subtracted from their respective measurements. The reaction rates were calculated from the slope of the plot of absorbance versus time curve. Elastase inhibition was then calculated using the following equation:

Elastase inhibition (%) = [(A − B)/A] × 100,(1)

where A is the reaction rate of the enzyme-substrate mixture without the sample (after subtraction of the blank of the control) and B is the reaction rate in the presence of the sample (after subtraction of the blank of the sample). The half-maximal inhibitory concentration (IC_50_) was determined using nonlinear regression analysis with GraphPad Prism software (version 10.6.1, GraphPad Software, Boston, MA, USA). The evaluation of *H. coronarium* rhizome oils was performed in comparison with eucalyptol as a reference standard. Oleanolic acid was used as the positive control. All assessments were performed in triplicate.

#### 2.5.2. Hyaluronidase Inhibitory Activities

The hyaluronidase inhibitory activity of *H. coronarium* rhizome oils was evaluated following the method of Nema et al. (2011) [32]. Initially, hyaluronidase enzyme activity was determined and only the enzyme preparations exhibiting greater than 90% activity were utilized in the assay. Briefly, 20 μL of the sample was mixed with 100 μL of hyaluronidase solution (15 units/mL in phosphate buffer pH 6.5) and incubated at 37 °C for 10 min. Subsequently, 100 μL of hyaluronic acid solution (0.1 mg/mL in phosphate buffer pH 6.5) was added to initiate the enzymatic reaction. Following a further incubation at 37 °C for 45 min, 1 mL of acidic albumin solution (0.1% bovine serum albumin in 24 mM sodium acetate buffer, pH 3.75) was added and the mixture was incubated at room temperature for 10 min. To minimize potential assay interference caused by essential oil components (e.g., turbidity or intrinsic absorbance), both blank of sample (sample in buffer without enzyme and substrate) and blank of control (sample solvent in buffer without enzyme and substrate) were included and subtracted from their respective measurements. The absorbance of the resulting solution was measured at 600 nm using a multimode microplate reader (BMG Labtech GmbH, Ortenberg, Germany). Hyaluronidase inhibition was calculated using the following equation:

Hyaluronidase inhibition (%) = [(A − B)/A] × 100,(2)

where A is the absorbance of the enzyme-substrate mixture without the sample (after subtraction of the blank of the control) and B is the absorbance in the presence of the sample (after subtraction of the blank of the sample). The IC_50_ value was determined using nonlinear regression analysis with GraphPad Prism software (version 8.0.2, GraphPad Software, Boston, MA, USA). The evaluation of *H. coronarium* rhizome oils was performed in comparison with eucalyptol as a reference standard. EGCG was used as the positive control. All assessments were performed in triplicate.

#### 2.5.3. Anti-Tyrosinase Activities

The tyrosinase inhibitory activity of *H. coronarium* rhizome oils was evaluated following the methods of Saeio et al. (2011) [33]. Initially, tyrosinase enzyme activity was determined and only the enzyme preparations exhibiting greater than 90% activity were utilized in the assay. Briefly, 10 μL of the sample was mixed with 90 μL of 105 units/mL mushroom tyrosinase in phosphate buffer pH 6.5. After the incubation at room temperature for 5 min, 100 μL of substrate solution (either L-tyrosine or L-DOPA) was added, followed by a further incubation at room temperature for 30 min. To minimize potential assay interference caused by essential oil components (e.g., turbidity or intrinsic absorbance), both blank of sample (sample in buffer without enzyme and substrate) and blank of control (sample solvent in buffer without enzyme and substrate) were included and subtracted from their respective measurements. The absorbance was measured at 492 nm using a multimode microplate reader (BMG Labtech GmbH, Ortenberg, Germany). Tyrosinase inhibition was calculated using the following equation:

Tyrosinase inhibition (%) = [(A − B)/A] × 100,(3)

where A represents the absorbance of the reaction mixture without the sample (after subtraction of the blank of the control) and B represents the absorbance of the reaction mixture with the sample (after subtraction of the blank of the sample). The IC_50_ value was determined using nonlinear regression analysis with GraphPad Prism software (version 8.0.2, GraphPad Software, Boston, MA, USA). The evaluation of *H. coronarium* rhizome oils was performed in comparison with eucalyptol as a reference standard. Kojic acid was used as the positive control. All assessments were performed in triplicate.

### 2.6. Development of Encapsulated H. coronarium Essential Oil

The encapsulation of *H. coronarium* rhizome essential oils was developed using a freeze-drying method as described by Sahlan et al. (2019) and Todorović et al. (2022) with slight modifications [34,35]. Maltodextrin or gum Arabic was used for encapsulation at a concentration of 10% *w*/*w* based on the total emulsion system, as shown in Table 1. The effect of varying essential oil loading content on the encapsulated powders was also evaluated to assess their impact on the physicochemical properties, encapsulation efficiency, and overall stability of the resulting systems. Initially, the dried Maltodextrin or gum Arabic was dispersed in DI water at a concentration of 20% *w*/*v*. The dispersion was stirred at 40 °C using a magnetic stirrer (AM4, VELP Scientifica, Usmate Velate, Italy) for 30 min to ensure complete solubilization. The solution was then allowed to hydrate overnight at ambient temperature (approximately 25 °C). The following day, *H. coronarium* rhizome oil was added to the pre-hydrated Maltodextrin or gum Arabic at oil-to- encapsulating material ratios of 1:20, 3:20, and 5:20. The mixture was homogenized using a high-speed homogenizer (T25 digital Ultra-Turrax^®^, IKA-Werke, Baden-Württemberg, Germany) at 10,000 rpm for 5 min. The resulting mixture was immediately subjected to freeze-drying using a vacuum freeze-dryer (Kinetic Engineering Co., Ltd., Bangkok, Thailand) at −40 °C for 19 h under reduced pressure. The resulting encapsulated powders were carefully collected, sealed in airtight containers, and stored at 4 °C in the dark until further analysis.

### 2.7. Characterization of Encapsulated H. coronarium Essential Oil

#### 2.7.1. Morphology Analysis

Morphological analysis of the encapsulated *H. coronarium* essential oil was conducted using a field emission scanning electron microscope (TESCAN, Brno, Czech Republic). In brief, the samples were mounted onto specimen holders using double-sided conductive carbon tape, followed by gold coating via a carbon coater and high-vacuum sputter coater (Safematic CCU-010, Zizers, Switzerland). Imaging was performed at magnifications of 2000× and 10,000× to assess the surface morphology and microstructure of the encapsulates.

#### 2.7.2. Hygroscopicity

The hygroscopicity of each encapsulation system was evaluated following the method described by Mazumder et al. (2020) [36]. Briefly, 2 g of encapsulated powder was evenly spread in a petri dish and placed in a desiccator containing saturated sodium sulfate solution to maintain a controlled relative humidity of 80 ± 1%. The samples were weighed daily to monitor moisture uptake until the weight stabilized. Hygroscopicity was expressed as g of moisture absorbed per 100 g of dry solids and calculated using the following equation:

Hygroscopicity (g/100 g) = (A × 100)/(100 − (B/100)),(4)

where A is the final weight after drying and B is the moisture content of the sample, which was measured using a moisture analyzer (Mettler Toledo™ HC103, Greifensee, Switzerland). All assessments were performed in triplicate.

#### 2.7.3. Moisture Content

The moisture content of each encapsulation system was determined using a moisture analyzer (Mettler Toledo™ HC103, Greifensee, Switzerland). Briefly, 1 g of encapsulated powder was placed on an aluminum pan and dried at 105 °C until a constant weight was achieved. All assessments were performed in triplicate.

#### 2.7.4. Water Activity

The water activity of each encapsulation system was measured using a water activity meter (Novasina, Lachen, Switzerland). Briefly, 1 g of encapsulated powder was placed into the sample cup, and measurements were performed. All assessments were performed in triplicate.

### 2.8. Entrapment Efficiency (EE) Determination

EE of each encapsulation system was determined following the method described by Carneiro et al. (2013) [37]. Briefly, 1.5 g of encapsulated powder was mixed with 15 mL of hexane using a vortex mixer (Vortex Genie 2, Scientific Industries, Ocala, FL, USA) for 10 min at room temperature to extract the free oil that was non-encapsulated. The resulting mixture was then filtered through Whatman No. 1 filter paper. The powder residue on the filter was rinsed three times with 20 mL of hexane. The pooled hexane extracts were evaporated to constant weight and the mass of the residue was used to quantify the surface oil. The total oil content was assumed to be equivalent to the initial oil used in the encapsulation formulation. EE was calculated using the following equation:

EE (%) = [(A − B)/A] × 100,(5)

where A is the total oil content that was the initial oil used for encapsulation and B is the surface oil that was non-encapsulated oil extracted from the powder. All assessments were performed in triplicate.

### 2.9. Determination of the Biological Stability of Encapsulated H. coronarium Essential Oil

The biological stability of encapsulated *H. coronarium* essential oil was evaluated in comparison with the solution, using 50% *v*/*v* ethanol as the solvent. All samples were stored in well-closed containers, protected from light, and subjected to an accelerated heating–cooling stability test consisting of six cycles. In each cycle, samples were stored at 45 °C for 24 h, followed by storage at 4 °C for another 24 h. After completion of the six cycles, the formulations were evaluated for their biological activities, including anti-elastase, anti-hyaluronidase, and anti-tyrosinase activities (using L-tyrosine and L-DOPA as substrates), as described in Section 2.5. Each sample was dissolved or, in the case of the solution, diluted in ethanol to a concentration of 1% *w*/*w* prior to biological activity assessment. The remaining inhibitory activity was calculated relative to the initial activity prior to the stability test using the following equation:

Remaining activity (%) = A/B × 100,(6)

where A is the percentage of inhibition after the accelerated stability test and B is the percentage of inhibition before the accelerated stability test. All assessments were performed in triplicate.

### 2.10. Irritation Test of Encapsulated H. coronarium Essential Oil

The irritation potential of encapsulated *H. coronarium* essential oil was evaluated using the hen’s egg test on the chorioallantoic membrane (HET-CAM) assay, an established alternative to animal testing that does not require ethical approval when performed within the first half of the embryonic incubation period [38,39]. Irritation was assessed based on three parameters, i.e., hemorrhage (leakage of blood from the vasculature), lysis (disappearance of small blood vessels due to rupture or degradation), and coagulation (formation of intravascular clots). The irritation score (IS) was calculated as previously described [40]. All assessments were performed in triplicate.

### 2.11. Statistical Analysis

All data are presented as mean ± standard deviation (SD). Statistical differences among groups were assessed using a *t*-test or one-way ANOVA, followed by Tukey’s post hoc test. Analyses were performed using GraphPad Prism software (version 8.0.2, GraphPad Software, Boston, MA, USA). Differences were considered significant with *p* < 0.05.

## 3. Results and Discussion

### 3.1. H. coronarium Rhizome Essential Oils

**Figure 1:**
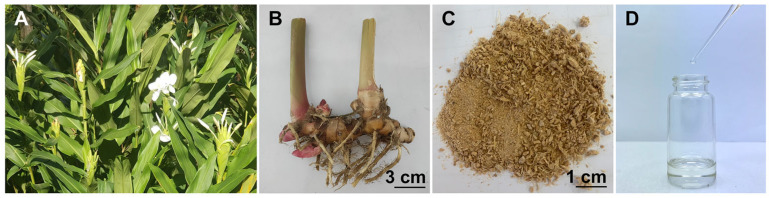
*H. coronarium* plants with blooming flowers (**A**), fresh rhizomes (**B**), dried rhizome powder (**C**), and essential oil (**D**).

**Figure 2:**
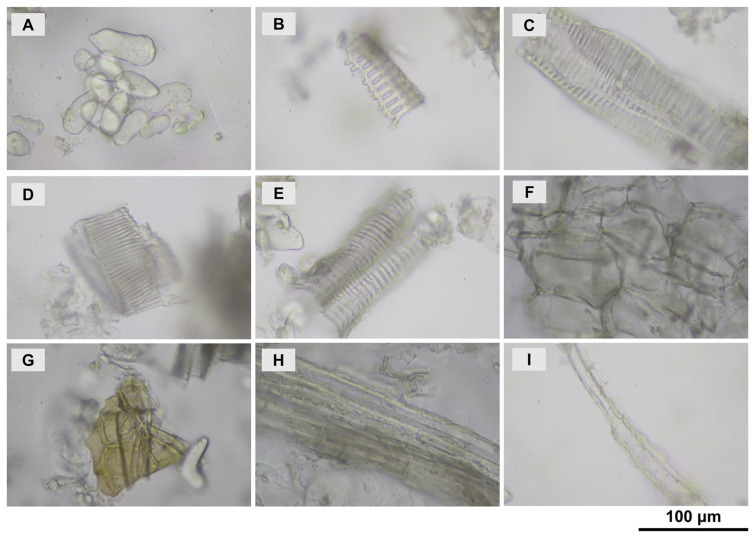
Microscope images of *H. coronarium* rhizome powder taken at 40× magnification, showing starch granules (**A**); part of a scalariform vessel (**B**); spiral vessels (**C**,**D**); a reticulated vessel (**E**); cork in oblique view (**F**); polygonal epidermis (**G**); a fiber bundle (**H**); and an isolated fiber (**I**).

*H. coronarium*, a widely cultivated tropical perennial herb (0.5–1.5 m tall), has aromatic, fleshy rhizomes rich in essential oils [28]. The morphology of the flowering plant and fresh rhizomes is shown in Figure 1A,B, respectively. The species is characterized by large, imbricated bracts, white tubular flowers, oblong leaves, and dense flower spikes [28,41]. Dried rhizome powder obtained after drying and grinding fresh rhizomes (Figure 1C) retained its aromatic properties and provided a suitable material for essential oil extraction [42]. Microscopic analysis of the dried rhizome powder (Figure 2) revealed abundant starch granules (Figure 2A), xylem elements including scalariform (Figure 2B), spiral (Figure 2C,D), and reticulated vessels (Figure 2E), as well as compact cork cells (Figure 2F), polygonal epidermal cells (Figure 2G), fiber bundles (Figure 2H), and isolated fibers (Figure 2I), consistent with previous reports for *H. coronarium* and other Zingiberaceae species [43,44]. The hydrodistilled rhizome essential oil (Figure 1D) appeared as a clear, pale-yellow liquid with an extraction yield of 0.25% *w*/*w* based on dried material.

Essential oil from *H. coronarium* rhizome has been reported to contain a variety of volatile compounds, with eucalyptol identified as a major constituent [27,45]. Other components commonly reported include β-pinene, 1,8-cineole (eucalyptol), and α-terpineol, which together contribute to the characteristic aroma and potential therapeutic properties of the oil [27,42,45]. The eucalyptol content of *H. coronarium* rhizome essential oil was found to be 31.4 ± 0.0% *w*/*w* in the current study, which is comparable to the 33.7% reported by Parida et al. (2015) [45]. However, it was lower than the 53.6 ± 0.5% *w*/*w* previously reported in our study using gas chromatography–mass spectrometry (GC-MS) analysis of the same oil sample [27]. The observed difference in eucalyptol content is likely a result of the distinct analytical approaches used, which have differing sensitivities. In this study, eucalyptol was quantified using HPLC-UV detection at 200 nm, corresponding to its maximum absorbance (λmax) [46], thereby enabling sensitive detection. Quantification was performed using an external standard calibration curve. In contrast, the previously reported GC-MS analysis expressed eucalyptol content as a relative percentage of the total volatile compounds detected, based on chromatographic peak area normalization [27]. These differences in analytical approach and quantification methodology may contribute to the observed variation in eucalyptol content. However, it should be clearly acknowledged that the discrepancy between the HPLC-UV result (31.4 ± 0.0% *w*/*w*) and the previously reported GC-MS value (53.6 ± 0.5% *w*/*w*) is relatively large and represents a limitation of the analytical approach. Future studies employing complementary or standardized analytical methods are recommended to achieve more consistent and comparable quantification. Despite this variation, eucalyptol is consistently identified as a major constituent of the rhizome essential oil, highlighting its key role in contributing to the characteristic aroma of *H. coronarium* and reinforcing its potential value for applications in fragrances, cosmetics, and other industries. Accordingly, the present study focused on the targeted quantification of this key marker compound using HPLC-UV, while comprehensive compositional profiling was not performed since it had been previously established by GC-MS analysis [27].

### 3.2. Biological Activities of H. coronarium Rhizome Essential Oils

**Figure 3:**
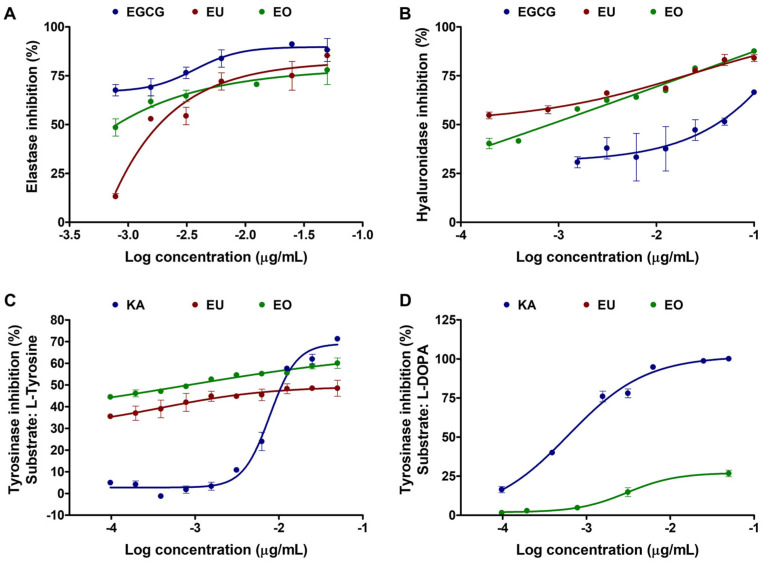
Dose–response curves of epigallocatechin gallate (EGCG), kojic acid (KA), eucalyptol (EU), and *H. coronarium* rhizome essential oil (EO) showing their inhibitory activities against elastase (**A**), hyaluronidase (**B**), and tyrosinase, using L-tyrosine (**C**) and L-DOPA (**D**) as substrates.

The biological activities of *H. coronarium* rhizome essential oil, in comparison with those of eucalyptol, its major constituent, are shown as dose–response curves in Figure 3, with the corresponding IC_50_ values summarized in Table 2. The findings indicated that both essential oil and eucalyptol exhibited significant enzyme inhibitory activities, although their potencies varied depending on the enzyme type. For anti-elastase activity (Figure 3A), *H. coronarium* rhizome essential oil exhibited strong inhibitory potential, with an IC_50_ value of 0.5 ± 0.0 µg/mL, which was stronger than that of pure eucalyptol (IC_50_ value of 2.8 ± 0.0 µg/mL). These results indicated that the essential oil possessed a more potent elastase-inhibitory effect than its major constituent alone. The enhanced activity may be attributed to additive or even synergistic effects among the various terpenoid components present in the oil, which could collectively contribute to its greater enzyme inhibition. A similar effect has been previously reported by Abdulazeez et al. (2025), who suggested that limonene exhibited enhanced biological activity, including inhibition of elastase, when combined with other monoterpenes through synergistic or additive interactions rather than when used alone [47]. Several studies have reported that essential oils in which eucalyptol is a major constituent exhibit significant elastase inhibition [48,49,50], suggesting that eucalyptol contributes to elastase inhibitory activity. However, no clear IC_50_ value for the pure compound has been reported. An essential oil from *Mentha viridis*, which contains approximately 11.8% eucalyptol, showed elastase inhibition with an IC_50_ of 114.24 ± 1.22 µg/mL [49]. Another essential oil, derived from Illicium anisatum and containing 36.7% eucalyptol, exhibited elastase inhibition with an IC_50_ value of about 1.79 mg/mL [50]. In addition, Mady et al. (2024) reported the anti-elastase potential of eucalyptol, identified as the major constituent of *Melaleuca rugulosa* (Link) Craven leaves, which also exhibited potent anti-elastase activity [51]. Although elastase inhibition cannot be attributed to eucalyptol alone, the available evidence suggests that eucalyptol contributed significantly to the anti-elastase activity of essential oils rich in this compound, including *H. coronarium*. The strong anti-elastase activity suggests that *H. coronarium* rhizome essential oil may serve as a promising natural source of elastase inhibitors, potentially beneficial for anti-aging or skin-protective applications.

Interestingly, the *H. coronarium* rhizome essential oil exhibited the strongest anti-hyaluronidase activity, with an IC_50_ value of 0.1 ± 0.0 µg/mL, which was stronger than that of eucalyptol (0.8 ± 0.0 µg/mL) and substantially stronger than EGCG, which served as the positive control (16.4 ± 0.0 µg/mL). Although data on the IC_50_ of pure eucalyptol for anti-hyaluronidase activity are limited, Rini et al. (2012) reported a value of 1.17 mg/mL [52]. The much lower IC_50_ of eucalyptol in the current study compared to Rini et al. (2012) likely reflected differences in assay conditions and data analysis, as Rini et al. (2012) estimated IC_50_ from only three concentrations (1, 2, and 4 mg/mL), providing a rough approximation that may overestimate inhibitory potency [52]. However, the present study confirmed and extended the findings of our previous work on *H. coronarium* aromatic extracts by further evaluating their activities in a dose–response manner, determining IC_50_ values, and comparing them with those of the major constituent, eucalyptol, to better understand the contribution of individual compounds to the observed biological effects [27]. Consistent with earlier results, the rhizome-derived essential oil exhibited strong anti-hyaluronidase properties, indicating its potential in anti-aging applications [27]. The current study further demonstrated that the rhizome essential oil exerted significant anti-hyaluronidase activity, with IC_50_ values lower than those of eucalyptol and the standard reference compounds ECGC, suggesting promising applications for the prevention of wrinkle formation. The pronounced inhibitory activity of the essential oil may be attributed to synergistic interactions among multiple terpenoid constituents, where minor components contribute to enhanced overall activity beyond that of the major compound alone [51,53,54]. Additional studies employing complementary assay methods and isolation of active constituents would be valuable to further validate the observed inhibitory effects and clarify the underlying mechanisms. As hyaluronidase is an enzyme that degrades hyaluronic acid, a key component of the extracellular matrix (ECM) that maintains skin hydration, elasticity, and structural integrity, the inhibition of this enzyme could help preserve hyaluronic acid levels, maintain ECM integrity, and prevent skin aging, including the formation of wrinkles and loss of firmness [55,56]. Excessive hyaluronidase activity disrupts the ECM, leading to reduced skin firmness, loss of elasticity, and wrinkle formation, all of which are hallmarks of skin aging [55]. In cosmetic applications, controlling hyaluronidase activity is critical for preventing premature skin aging and preserving hyaluronic acid content in the skin [55,57]. Hyaluronidase inhibitors are therefore considered promising anti-aging agents due to their potential to maintain hyaluronic acid homeostasis and protect against ECM degradation [55]. Additionally, hyaluronidase activity affected the longevity and effectiveness of hyaluronic acid fillers used in esthetic dermatology, highlighting the importance of understanding its function in skin physiology [57]. Therefore, the *H. coronarium* rhizome essential oil, with potent anti-hyaluronidase activity, represents a promising candidate for both anti-aging skincare strategies and cosmetic applications.

Aside from the potential of *H. coronarium* rhizome essential oil for anti-skin aging, skin whitening can be achieved by modulating tyrosinase activity. Tyrosinase is a key enzyme in melanogenesis that exhibits two distinct catalytic activities, including monophenolase, converting L-tyrosine to L-DOPA, and diphenolase, oxidizing L-DOPA to dopaquinone [58]. In the present study, the essential oil demonstrated promising anti-tyrosinase activity against both substrates, with IC_50_ values of 1.9 ± 0.0 µg/mL for L-tyrosine (Figure 3C) and 595.4 ± 0.3 µg/mL for L-DOPA (Figure 3D). Eucalyptol was identified as a major constituent responsible for the anti-tyrosinase activity when L-tyrosine was used as the substrate, but not for L-DOPA. The findings relied on a previous study indicating that eucalyptol (1,8-cineole), the major component of Eucalyptus globulus essential oil, exhibited weak monophenolase inhibition, while no diphenolase inhibition was observed [59]. Eucalyptol was suggested to inhibit tyrosinase mainly via non-covalent binding and structural perturbation, rather than by direct catalytic suppression [59]. Eucalyptol likely exhibited an apparent substrate preference for L-tyrosine, as it more effectively interfered with tyrosinase’s monophenolase activity, which catalyzed the initial hydroxylation of L-tyrosine to L-DOPA [59]. This reaction required access to a relatively narrow and hydrophobic region adjacent to the dicopper catalytic center of the tyrosinase enzyme, a site with which eucalyptol would be structurally suited to interact [60]. In contrast, the subsequent diphenolase reaction, in which L-DOPA was oxidized to dopaquinone, relied heavily on the ability of the substrate to chelate copper ions within the active site [60]. Eucalyptol could not disrupt the diphenolase reaction due to its lack of phenolic or metal-chelating functional groups. Consequently, its inhibitory effect was more pronounced during the monophenolase reaction than during diphenolase catalysis, giving the functional impression of L-tyrosine-specific inhibition.

The IC_50_ value for tyrosinase inhibition of *H. coronarium* rhizome essential oil was significantly lower than that of both eucalyptol and kojic acid, which served as positive controls, particularly when L-tyrosine was used as the substrate, suggesting a more potent inhibitory effect. This indicated that the essential oil strongly inhibited tyrosinase’s monophenolase activity, the rate-limiting step in melanin biosynthesis [61]. In contrast, when L-DOPA was used as the substrate, representing diphenolase activity, the essential oil exhibited moderate inhibition, whereas eucalyptol itself showed no effect. These observations suggested that the minor components of the essential oil contribute to tyrosinase inhibition. This substrate-dependent efficacy reflected the distinct catalytic mechanisms of tyrosinase, in which monophenolase inhibition targeted the early stages of melanin formation, whereas diphenolase inhibition affected the oxidation of L-DOPA to dopaquinone, thereby influencing melanin polymerization [62]. Therefore, testing both substrates provides a more comprehensive assessment of anti-melanogenic potential, and the combination of eucalyptol with other minor compounds in the essential oil may offer broad-spectrum inhibition, enhancing its effectiveness as a skin-whitening agent.

### 3.3. Encapsulated H. coronarium Rhizome Essential Oils

**Figure 4:**
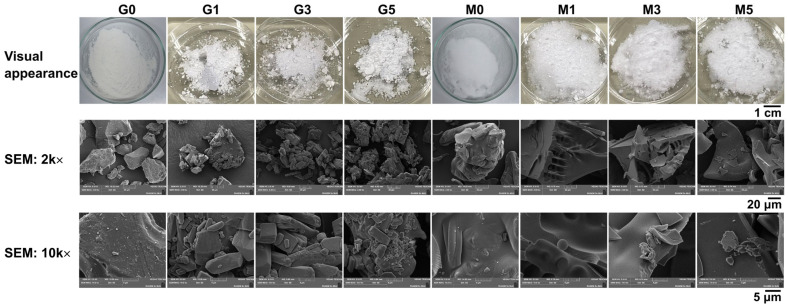
Visual appearance and scanning electron microscopy (SEM) images of encapsulated *H. coronarium* rhizome essential oil powders using gum Arabic (G) and maltodextrin (M) as encapsulating agents. G0 and M0 refer to the raw materials of gum Arabic and maltodextrin, respectively. The numbers 1, 3, and 5 indicate increasing essential oil loading concentrations (% *w*/*w*). SEM images are shown at magnifications of 2 k× (middle row) and 10 k× (bottom row) to illustrate the microstructure of the powders.

The encapsulation of *H. coronarium* rhizome essential oils using gum Arabic or maltodextrin as encapsulating agents, with essential oil loadings of 1, 3, and 5% *w*/*w*, is shown in Figure 4. Both gum Arabic and maltodextrin are widely employed carbohydrate-based encapsulating agents in essential oil encapsulation due to their complementary functional properties, including emulsification, film formation, and the ability to enhance particle stability and oil retention [63]. Gum Arabic, a natural polysaccharide, has been reported to possess emulsifying ability, high water solubility, and film-forming properties, which allow it to stabilize oil-in-water emulsions effectively and protect hydrophobic compounds, especially essential oils [64]. Visually, gum Arabic appears as a relatively fine powder. Under SEM observation, the particles exhibit a slightly irregular, non-uniform morphology and are less compact, typically showing a rough, fragmented texture with heterogeneous surface features (Figure 4). Maltodextrin, a partially hydrolyzed starch, is used due to its low viscosity, high solubility, and ability to form compact and uniform particles [64,65]. Visually, maltodextrin presents as a finer, smoother, and more uniform powder. Under SEM observation, the particles appear more compact, with relatively dense and continuous surfaces (Figure 4).

The raw polymers exhibit morphologies distinct from the encapsulated particles. The visual appearance of encapsulated *H. coronarium* rhizome essential oil powders varied slightly depending on the encapsulating agents and essential oil content. This observation suggested that microstructures are primarily governed by the encapsulation conditions rather than solely by the intrinsic properties of the raw materials. Encapsulated powders prepared with gum Arabic exhibited coarser, more irregular particles with porous surfaces, whereas those prepared with maltodextrin were finer, smoother, and more uniform. Increasing essential oil loading had minor effects on morphology but slightly increased surface irregularities. The gum Arabic encapsulation systems (G1, G3, G5) exhibited a relatively uniform texture across all concentrations, whereas the maltodextrin encapsulation systems (M1, M3, M5) formed larger agglomerates as the essential oil content increased. The likely explanation is the lower emulsifying and oil-retaining capabilities of maltodextrin compared to gum Arabic [66].

SEM confirmed that particles encapsulated with gum Arabic were rough, irregular, and aggregated, whereas those encapsulated with maltodextrin displayed smoother and more compact structures, suggesting that maltodextrin provides a more uniform encapsulation matrix than gum Arabic. At higher magnification (10 k×), particles encapsulated with gum Arabic exhibited heterogeneous, flaky, and porous surfaces, whereas those encapsulated with maltodextrin showed relatively flat, plate-like structures with fewer irregularities. These differences in microstructure indicated that the encapsulating agents strongly influenced morphology and potentially the stability of the encapsulated oil. The encapsulating agent, with its inherently porous structure, possesses a large specific surface area and excellent adsorption performance, thereby enhancing oil adsorption, whereas a highly compact structure might hinder release [67]. In addition, the interconnected porous network facilitates diffusion-controlled transport, promoting sustained release behavior, as suggested by Sun et al. (2021) [68]. Across both encapsulation matrices, varying the essential oil content from 1% to 5% *w*/*w* slightly altered the microstructure, with higher oil loads leading to increased surface roughness and minimal pore formation. For further quality control, it is recommended to evaluate particle size and size distribution, as these parameters can impact stability, release behavior, and reproducibility of the encapsulated formulations.

The physicochemical properties, including hygroscopicity, water activity, moisture content, and entrapment efficiency of encapsulated *H. coronarium* rhizome essential oil powders prepared with gum Arabic (G1, G3, G5) and maltodextrin (M1, M3, M5), are summarized in Table 3.

Hygroscopicity, an important parameter indicating a powder’s tendency to absorb moisture, which can affect its flowability and storage stability, was also evaluated. It was noted that the hygroscopicity varied markedly between the two encapsulating agents. Maltodextrin-encapsulated powders exhibited significantly higher hygroscopicity (0.19–0.21%) compared with gum Arabic–encapsulated powders (0.03–0.06%) (*p* < 0.05). These findings can be attributed to the fact that maltodextrin produced finer encapsulated powders compared with gum Arabic (Figure 4). Finer particles generally exhibit higher hygroscopicity, as a smaller particle size provides a larger surface area, which enhances the tendency to absorb water from the environment [69]. In addition, gum Arabic has been reported to possess compact and highly branched polysaccharide structures, which limit moisture uptake [70,71]. The lower hygroscopicity of the gum Arabic–based encapsulation system suggested better physical stability during storage. However, the hygroscopicity of both encapsulation systems remained very low and within the non-hygroscopic range (<10%), according to the criteria of Pisecky (2012) [72], indicating acceptable moisture-handling properties for practical applications despite the differences between encapsulating agents.

Similarly, moisture content also varied depending on both the encapsulating agents and essential oil loading. Maltodextrin powders contained higher moisture contents (4.3–6.3%), than gum Arabic powders (2.0–2.3%). Moisture content is a major factor influencing the stability of encapsulating powders, and low moisture content combined with controlled hygroscopicity are desirable physicochemical characteristics [73]. Maintaining moisture content below 5% is particularly important, as it creates unfavorable conditions for microbial growth, enhances overall stability, and extends shelf life [74]. Low moisture content also facilitates handling, storage, and potential use of the powder in various applications, making it a critical parameter for ensuring product quality and functionality [73]. The results indicated that gum Arabic-based encapsulations maintained moisture content within acceptable limits, providing favorable conditions for powder stability and an extended shelf life.

However, distinct differences in water activity were also observed in the encapsulation systems with different encapsulating agents. Gum Arabic encapsulated powders showed significantly higher water activity values (0.40–0.43), whereas maltodextrin encapsulated powders had lower water activity (0.26–0.31) (*p* < 0.05). Water activity is a key indicator of microbial and chemical stability [75,76]. Interestingly, although maltodextrin-based powders exhibited higher hygroscopicity (due to their smaller particle size and larger surface area), they demonstrated lower water activity compared with gum Arabic powders. This apparent contradiction can be explained by the strong water-binding capacity of maltodextrin [77]. Although the powder readily absorbs moisture from the environment, much of this water is tightly bound to the polymer chains and therefore unavailable as free water. The reduced water activity in maltodextrin-based powders may support improved microbial and chemical stability, although their higher hygroscopicity could still pose handling challenges under humid conditions.

Entrapment efficiency is a primary measure of encapsulation success, indicating how effectively the essential oil is retained within the matrix to ensure controlled release, protection, and long-term stability [23]. The current study demonstrated that the entrapment efficiency was uniformly high across all formulations (99.8–99.9%), with no significant differences observed among samples. These findings indicate that both gum Arabic and maltodextrin can effectively encapsulate essential oils at the loading levels used. These results are consistent with a study by Cortés-Rojas et al. (2014) [78], which reported that freeze-drying can better stabilize extracts than spray-drying because it avoids the use of high temperatures, thereby significantly reducing the loss of volatile compounds [78]. However, it should be noted that the total oil content was estimated based on the initial amount added, which may introduce some uncertainty due to potential volatilization during processing. The use of the initially added oil as the total oil content may lead to a slight overestimation of encapsulation efficiency, as potential losses during processing are not accounted for. Therefore, further studies involving direct quantification of retained oil content or evaluation of retained biological activities after stability testing are recommended to confirm these findings.

### 3.4. Biological Stability of Encapsulated H. coronarium Rhizome Essential Oils

**Figure 5:**
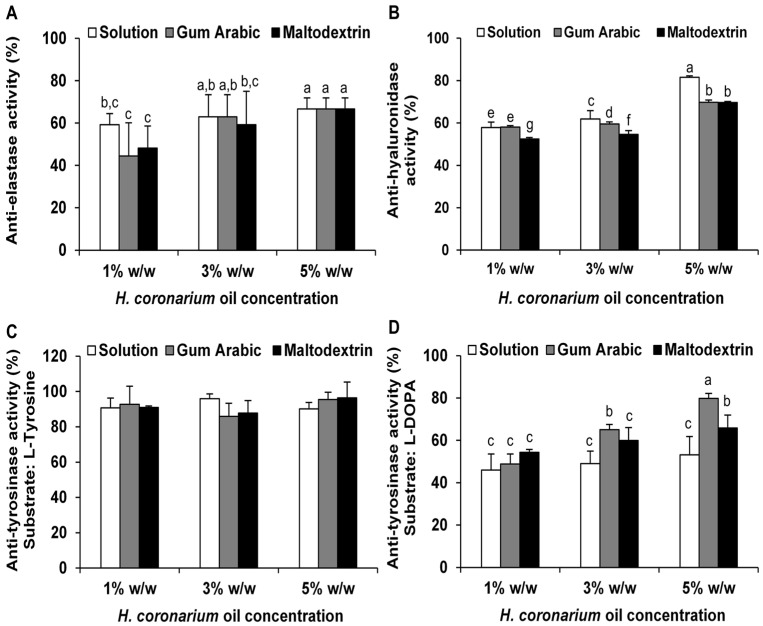
Remaining inhibitory activities against elastase (**A**), hyaluronidase (**B**), and tyrosinase using L-tyrosine (**C**) and L-DOPA (**D**) as substrates of *H. coronarium* rhizome essential oil in solution form (white bars), and in encapsulated powder form using gum Arabic (gray bars) and maltodextrin (black bars), with results expressed as remaining activity relative to the initial activity (before the test). Data are expressed as mean ± SD. Different superscript letters indicate significant differences (*p* < 0.05).

The stability of enzyme inhibitory activities of *H. coronarium* rhizome essential oil is shown in Figure 5 after an accelerated stability test using six heating–cooling cycles. Generally, essential oils are inherently unstable, volatile compounds that are highly susceptible to degradation when exposed to external factors such as light, heat, oxidation, and evaporation [79]. Accordingly, the solution of *H. coronarium* rhizome essential oil exhibited a marked reduction in biological activity, particularly at low concentrations of the oil, with anti-elastase activity decreasing to 76.5 ± 3.0%, anti-tyrosinase activity (using L-DOPA as substrate) to 64.1 ± 12.2%, and anti-hyaluronidase activity to 69.3 ± 1.8%. In contrast, encapsulated formulations exhibited significantly higher remaining activities compared with the solution across all activities, confirming that encapsulation effectively protects bioactive compounds against thermal fluctuation and stress. The greatest loss in biological activities of the solution is likely due to the direct exposure of volatile and thermolabile constituents to heat, oxygen, and repeated phase changes, leading to degradation and evaporation [79,80]. In contrast, both gum Arabic and maltodextrin encapsulated powders maintained high levels of inhibitory activity, mostly above 90% remaining activity, demonstrating the protective role of the carrier matrix. The benefit of encapsulation lies in its ability to reduce the volatilization of essential oil components and provide a physical barrier against heat, thereby enhancing the overall stability of bioactive compounds [81]. This results in improved retention of functional activity after stress conditions. The retained biological stability of the essential oil in the encapsulation systems may reflect its effective retention within the matrix, consistent with the high entrapment efficiency observed (99.8–99.9%), as shown in Table 3. This suggested that the encapsulation system could effectively limit volatilization and protect sensitive constituents from environmental degradation. Such protection helped preserve the functional bioactivity of the essential oil over time. However, it should be noted that the entrapment efficiency was calculated based on the initially added oil content rather than an experimentally determined total oil content, which may lead to slight overestimation.

The findings indicated that both gum Arabic and maltodextrin encapsulated powders exhibited comparable results (Figure 5). However, maltodextrin showed a slight tendency to provide greater stability than gum Arabic. This may be attributed to its ability to form a dense and less porous matrix [82,83], which more effectively entraps *H. coronarium* rhizome essential oil and restricts its evaporation. In contrast, gum Arabic tends to form a more porous and less compact structure, which may allow greater diffusion of essential oil from the matrix [67,68], resulting in slightly lower protective efficiency. Therefore, encapsulation, particularly with maltodextrin, significantly improved the stability of *H. coronarium* essential oil compared to the solution, making it a promising approach for maintaining bioactivity in cosmetic and pharmaceutical applications. Furthermore, it could be used as an intermediate delivery system and subsequently incorporated into suitable topical formulations.

### 3.5. Irritation of Encapsulated H. coronarium Rhizome Essential Oils

**Figure 6:**
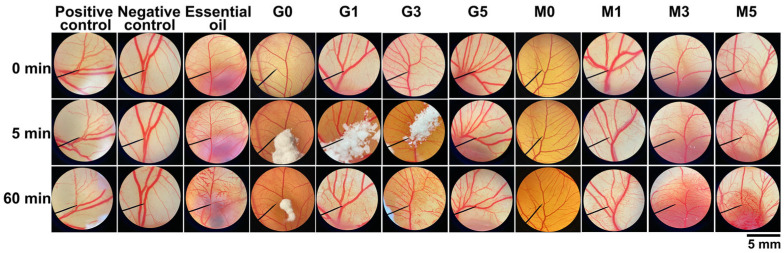
Photographs of the chorioallantoic membrane (CAM) before sample application (top row) and after 5 min (middle row) and 60 min (bottom row) of exposure to encapsulated *H. coronarium* rhizome essential oil powders using gum Arabic (G) or maltodextrin (M) as encapsulating agents. G0 and M0 refer to the raw materials of gum Arabic and maltodextrin, respectively. Numbers 1, 3, and 5 indicate increasing essential oil loading concentrations (% *w*/*w*). Positive control: 1% *w*/*v* sodium lauryl sulfate (SLS); negative control: 0.9% *w*/*v* sodium chloride (NaCl) solution.

The irritation potential of *H. coronarium* rhizome oil, its encapsulated powders, and the blank polymer matrices was evaluated using the HET-CAM assay, with visual observations shown in Figure 6 and calculated irritation score presented in Table 4. The results of the primary skin irritation test showed clear differences in irritation potential among the evaluated samples. The positive control (1% *w*/*v* SLS) produced a high irritation score of 16.4 ± 3.6, which corresponds to severe irritation and confirms the sensitivity of the assay. In contrast, the negative control (0.9% *w*/*v* NaCl) produced an irritation score of 0.0 ± 0.0, indicating no irritation and validating the reliability of the experimental conditions. The blank polymer matrices, both gum Arabic and maltodextrin, showed no irritation, indicating they are safe. The *H. coronarium* rhizome oil exhibited a similarly high irritation score (13.3 ± 1.4), also classified as severe irritation. This strong irritant effect is consistent with widely reported characteristics of essential oils, which often contain volatile terpenoids and aldehydes known to disrupt the integrity of the stratum corneum and cause irritations [20,84]. In comparison, all formulated samples demonstrated a dramatic reduction in irritation potential. The encapsulated powders using gum Arabic (G1, G3, and G5) showed irritation scores between 3.9 ± 1.1 and 4.1 ± 0.7, while those formulated with maltodextrin (M1, M3, and M5) exhibited similarly low irritation values ranging from 3.6 ± 0.3 to 4.2 ± 1.4. Despite minor variation among the formulations, all samples were consistently classified as mildly irritating, with the irritation score between 1.0 and 4.9 [40]. The statistically significant differences between the *H. coronarium* rhizome oil and its encapsulated powders confirmed that the formulation process effectively reduced irritation. These findings suggest that the encapsulating agents acted as a protective barrier, thereby limiting the direct contact of irritant constituents with the CAM and indicating its potential suitability for further application to the skin. Such systems may also modulate the release of volatile compounds, thereby reducing their immediate impact on cutaneous tissues [85]. Although the findings from the HET-CAM test exhibited mild irritation, the encapsulations may still be considered safe for skin, as the CAM models eye tissue and mucous membranes, which are more sensitive than skin [86]. While the native essential oil demonstrated severe irritation, incorporation into the encapsulations significantly improved skin compatibility. These results support the suitability of these encapsulated powders for potential cosmetic or dermatological use.

## 4. Conclusions

This study provided the evidence supporting the potential of *H. coronarium* rhizome essential oil as a valuable bioactive ingredient for cosmetic and dermatological applications. Eucalyptol was consistently identified as the major constituent of the rhizome essential oil and contributed substantially to its biological properties. The essential oil exhibited potent anti-elastase, anti-hyaluronidase, and anti-tyrosinase activities, with IC_50_ values markedly lower than those of eucalyptol alone and, in some cases, lower than standard positive controls. Notably, the essential oil demonstrated exceptionally strong anti-hyaluronidase activity, surpassing both eucalyptol and EGCG, reflecting synergistic interactions among its terpenoid components. These biological activities highlighted its strong potential for anti-aging and skin-whitening applications through mechanisms involving the preservation of extracellular matrix integrity and modulation of melanogenesis. Nevertheless, further studies are warranted to validate these effects and confirm their efficacy and safety in practical applications. Additionally, encapsulation with gum Arabic or maltodextrin produced highly water-soluble powders with excellent EE (>99.8%), distinct microstructures, and physicochemical properties suitable for incorporation into hydrophilic cosmetic systems. However, the EE values were calculated based on the initially added oil content, which may result in slight overestimation. Moreover, both encapsulated powders significantly enhanced biological stability, retaining high enzyme inhibitory activities (>90%) after accelerated heating–cooling cycles compared with the ethanolic solution. The improved stability likely reflects effective retention of the essential oil within the encapsulation matrix. Based on the physicochemical, morphological, and irritation data, encapsulation of the essential oil was successful at loadings up to 5% *w*/*w*. Gum Arabic-based matrices were preferred when low moisture uptake and handling stability are priorities, while maltodextrin provided lower water activity and finer powders. In addition, both encapsulating agents significantly reduced the irritation potential of the native essential oil, as evidenced by markedly lower HET-CAM irritation scores. These findings confirmed that encapsulation effectively limited direct contact between irritant terpenoids and biological membranes, thereby improving dermal compatibility. Therefore, the results highlighted the promise of *H. coronarium* rhizome essential oil as a multifunctional bioactive ingredient and demonstrate that encapsulation can markedly enhance its stability, safety, and usability. The resulting encapsulated powder can be utilized either as a standalone intermediate delivery system or preferably incorporated into suitable topical formulations, depending on the intended application. The compatibility of the encapsulated powders with cosmetic bases is therefore an important consideration. Further investigations into clinical safety and efficacy would support the advancement of these encapsulated powders toward commercial applications.

## Figures and Tables

**Table 1:** Composition of *H. coronarium* rhizome essential oils encapsulated powders.

Ingredients	Concentration (% *w*/*w*)					
	G1	G3	G5	M1	M3	M5
Essential oil	1	3	5	1	3	5
Gum Arabic	10	10	10	0	0	0
Maltodextrin	0	0	0	10	10	10
DI water	89	87	85	89	87	85

NOTE: Percentages refer to the total emulsion system (% *w*/*w* of each component relative to the total formulation, including polymer and water).

**Table 2:** Biological activities of *H. coronarium* rhizome essential oil and eucalyptol.

Ingredients	IC_50_ (µg/mL)			
	Anti-Elastase Activity	Anti-Hyaluronidase Activity	Anti-Tyrosinase Activity	
			L-Tyrosine	L-DOPA
EGCG	0.19 ± 0.04 ^a^	16.43 ± 0.04 ^c^	N/A	N/A
Kojic acid	N/A	N/A	15.10 ± 0.02 ^b^	0.59 ± 0.01 ^a^
Eucalyptol	2.76 ± 0.03 ^c^	0.83 ± 0.01 ^b^	18.10 ± 0.04 ^c^	ND
Essential oil	0.50 ± 0.03 ^b^	0.17 ± 0.03 ^a^	1.88 ± 0.01 ^a^	595.37 ± 0.26 ^b^

NOTE: IC_50_ = half-maximal inhibitory concentration; N/A = not available (test not performed); ND = not determined (tested but no activity observed). The letters a, b, and c indicate statistically significant differences among the tested compounds (*p* < 0.05).

**Table 3:** Physicochemical properties of encapsulated *H. coronarium* rhizome essential oils.

Formulation	Hygroscopicity (%)	Moisture Content (%)	Water Activity	Entrapment Efficiency (%)
G1	0.06 ± 0.01 ^c^	2.0 ± 0.1 ^d^	0.41 ± 0.00 ^c^	99.9 ± 0.0
G3	0.03 ± 0.01 ^b^	2.3 ± 0.1 ^d^	0.40 ± 0.00 ^c^	99.9 ± 0.1
G5	0.04 ± 0.01 ^b,c^	2.0 ± 0.4 ^d^	0.43 ± 0.02 ^c^	99.9 ± 0.0
M1	0.21 ± 0.00 ^a^	4.3 ± 0.5 ^c^	0.26 ± 0.00 ^b^	99.9 ± 0.1
M3	0.20 ± 0.01 ^a^	6.3 ± 0.2 ^a^	0.31 ± 0.00 ^a^	99.9 ± 0.0
M5	0.19 ± 0.01 ^a^	5.3 ± 0.3 ^b^	0.28 ± 0.02 ^a^	99.8 ± 0.3

NOTE: Different letters (a, b, c, and d) denote statistically significant differences among the encapsulated powders as determined by one-way ANOVA followed by Tukey’s post hoc test (*p* < 0.05).

**Table 4:** Irritation scores of *H. coronarium* rhizome oil and its encapsulated powders.

Samples	Irritation Score	Classification
Positive control (1% *w*/*v* SLS)	16.4 ± 3.6 ^a^	Severe irritation
Negative control (0.9% *w*/*v* NaCl)	0.0 ± 0.0 ^c^	No irritation
*H. coronarium* rhizome oils	13.3 ± 1.4 ^a^	Severe irritation
G1	4.1 ± 0.7 ^b^	Mild irritation
G3	3.9 ± 1.1 ^b^	Mild irritation
G5	4.1 ± 0.2 ^b^	Mild irritation
M1	3.9 ± 0.9 ^b^	Mild irritation
M3	4.2 ± 1.4 ^b^	Mild irritation
M5	3.6 ± 0.3 ^b,c^	Mild irritation

NOTE: SLS = sodium lauryl sulfate; NaCl = sodium chloride. Different letters (a, b, and c) denote statistically significant differences among the encapsulated powders as determined by one-way ANOVA followed by Tukey’s post hoc test (*p* < 0.05).

## Data Availability

The original contributions presented in this study are included in the article. Further inquiries can be directed to the corresponding author.

## Abbreviations

The following abbreviations are used in this manuscript:

AAAPVN	N-succinyl-Ala-Ala-Ala-p-nitroanilide
ANOVA	Analysis of variance
BSA	Bovine serum albumin
CAM	Chorioallantoic membrane
DI	Deionized
DMSO	Dimethyl sulfoxide
EC	Enzyme Commission number
EE	Entrapment efficiency
EGCG	Epigallocatechin gallate
EO	Essential oil
EU	Eucalyptol
GC–MS	Gas chromatography–mass spectrometry
HET-CAM	Hen’s egg test on the chorioallantoic membrane
HPLC	High-performance liquid chromatography
IC_50_	Half-maximal inhibitory concentration
ICH	International Council for Harmonisation
IS	Irritation score
KA	Kojic acid
L-DOPA	L-3,4-dihydroxyphenylalanine
LOD	Limit of detection
LOQ	Limit of quantification
MD	Maltodextrin
NaCl	Sodium chloride
R^2^	Coefficient of determination
SD	Standard deviation
SDGs	Sustainable Development Goals
SEM	Scanning electron microscopy
SLS	Sodium lauryl sulfate
TEA	Triethanolamine
Tris	Tris(hydroxymethyl)aminomethane
UV	Ultraviolet
